# Family planning in a rural setting in Uganda, the USHAPE initiative

**DOI:** 10.1080/17571472.2016.1241302

**Published:** 2016-10-16

**Authors:** Emily Clark, Clare Goodhart

**Affiliations:** ^a^ RCGP Junior International Committee, London, UK; ^b^ Lensfield Medical Practice, East of England, UK

**Keywords:** Family planning, family planning training, reproductive health services, volunteerism, capacity building

## Abstract

**Background:**

The total fertility rate in Uganda is 5.9 children per woman, and women admit to having nearly two more children than they actually want. The maternal mortality rate remains stubbornly high. Family planning saves lives. It prevents maternal deaths by delaying motherhood, helping women limit their family size and avoid unwanted pregnancies. It also reduces infant mortality.

**Setting:**

USHAPE (Ugandan Sexual Health and Pastoral Education) is an initiative run in conjunction with the Royal College of General Practitioners in south-west Uganda. USHAPE aims to disseminate positive messages about modern contraception in an attempt to dispel fears and misconceptions and address the high rate of unmet need.

**Question:**

The aim was to determine the rate of unmet need for family planning among women of reproductive age in the population local of Kisiizi hospital and to use the successful USHAPE model to train health workers to address this need.

**Methods:**

100 patients were screened in the outpatient department to determine the level of unmet need by asking 2 questions. Level 1 training aims enhance every staff member’s knowledge, so that the responsibility for family planning is adopted by the whole institution. Level 2 trains clinicians to become full family planning providers, with the necessary communication, educational and practical skills.

**Results:**

The screening for unmet need for contraception revealed that 51% have an unmet need, which is higher than the national average of 38%. Sixty-eight members of staff at Kisiizi trained to a basic level and a further 32 staff have been trained to Level 2 higher level.

**Conclusions/Discussion:**

The USHAPE approach has begun to tackle some of the barriers to accessing family planning, but there are further areas which need development. Our cascade model of training, involves training Ugandan USHAPE trainers with the aim of future scale up and long-term development.

## Key message

• Family planning saves lives but there are many cultural, religious, social and practical barriers to women accessing family planning in south-west Uganda.
• USHAPE aims to disseminate positive messages about modern contraception in an attempt to dispel fears and misconceptions and address the high rate of unmet need for family planning in south-west Uganda.
• USHAPE works by both screening for unmet need and training health workers in family planning.
• Timely input of short-term projects with a longer term endeavour is helpful.

## Why this matters to me

I first became interested in the low uptake of family planning in Uganda, when I volunteered with the Maternal and Newborn Hub from January to July 2015 at Kisiizi Hospital in a remote corner of south-west Uganda. Every day I witnessed the tragic stories of unwanted pregnancies, unsafe abortions and children suffering with malnutrition because their parents couldn’t afford to feed them. I wanted to find some way that I, as a family doctor and an outsider, could positively work with the local people to tackle a problem that was heavily entrenched in a web of cultural, social, gender and religious values.

The total fertility rate in Uganda is 5.9 children per woman [1], and women admit to having nearly two more children than they actually want.[2] The maternal mortality rate remains stubbornly high, with 25% of these deaths due to unsafe abortions.

Family planning prevents maternal deaths for a number of reasons. Firstly, by delaying motherhood, because adolescents are twice as likely to die in childbirth; national statistics show that by 18–19 years, 77% of Ugandan girls are sexually active, and the teenage pregnancy rate is 24%, highest in Africa.[3] Secondly, by helping women limit their family size and thereby avoid the more risky pregnancies as they get older. Thirdly, by avoiding unwanted pregnancies, which lead women to undergo illegal and dangerous abortions. Family planning also reduces infant mortality, because it is known that children born less than 2 years after their sibling are twice as likely to die.

Family planning is included in the 1978 World Health Organization Declaration of Alma Ata which defines Primary Health care as

essential health care based on practical, scientifically sound, and socially acceptable methods and technology made universally accessible to individuals and families in the community through their full participation and at a cost that the community and country can afford to maintain at every stage of their development in the spirit of self-reliance and self-determination.[4]

Family planning is the essential link to achieving many of the health related U.N. sustainable development goals.

USHAPE (Ugandan Sexual Health and Pastoral Education) is an initiative run in conjunction with the Royal College of General Practitioners delivering Family Planning and Sexual Health Training and Education in Uganda. Our work began at Bwindi Community Hospital in 2013, and we have since been awarded funding and support from the Tropical Health & Education Trust (THET), which has been a huge help in advancing our programmes. We hope to feedback lessons learned from our experiences in Uganda to inform the further development of WHO and USAID resources for family planning training. Bwindi Community Hospital was featured as a ‘Success Story’ on the WHO Family Planning training website.[5] With the support of Dr. Clare Goodhart and Bwindi hospital, I was able to take the initiative to Kisiizi Hospital in May 2015. USHAPE aims to disseminate positive messages about modern contraception in an attempt to dispel fears and misconceptions and address the high rate of unmet need for family planning in south-west Uganda [6].

## Aims

USHAPE works by both screening for unmet need and training health workers in family planning.

### Screening

The aim was to determine the rate of unmet need for family planning among women of reproductive age in the population local to Kisiizi hospital. A further aim was to begin to understand and work within the local cultural, religious, social and practical barriers to accessing family planning with the longer term goal of reducing the unmet need.

### Training

The aim was to use the successful USHAPE model [7] to train health workers in family planning, both to a basic Level 1, and key staff to a higher Level 2 which is equivalent to the UK Diploma in Sexual and Reproductive Health. A further aim was to train a Ugandan nurse as a trainer in family planning.

## Methods

### Screening

One hundred patients were screened in the outpatient department by asking the following questions.

*Are you on family planning?*

*Do you want a child in the next 2 years?*

If they answered no to both of these, they were classed as having an unmet need for family planning.

### Training

I attended training at Bwindi hospital so as to be able to successfully run Level 1 and Level 2 training at Kisiizi. Level 1 training aims to enhance the family planning knowledge of all hospital staff, so that the whole institution makes it part of their everyday work, rather than being left as the responsibility of the family planning nurse. Level 2 trains clinicians to become full family planning providers, with the necessary communication, educational and practical skills.

The author was also involved in the creation of a facilitation guide, so as to train a Ugandan nurse as a trainer.

## Results

### Screening

The screening for unmet need for contraception revealed that 51% have an unmet need, which is higher than the national average of 38%. In an average month, over 500 patients attending antenatal clinic, yet only 15 patients visiting the family planning clinic. There have already been improvements with 92 patients attending the family planning clinic in the month of August 2015.

### Training

**Figure 1.**
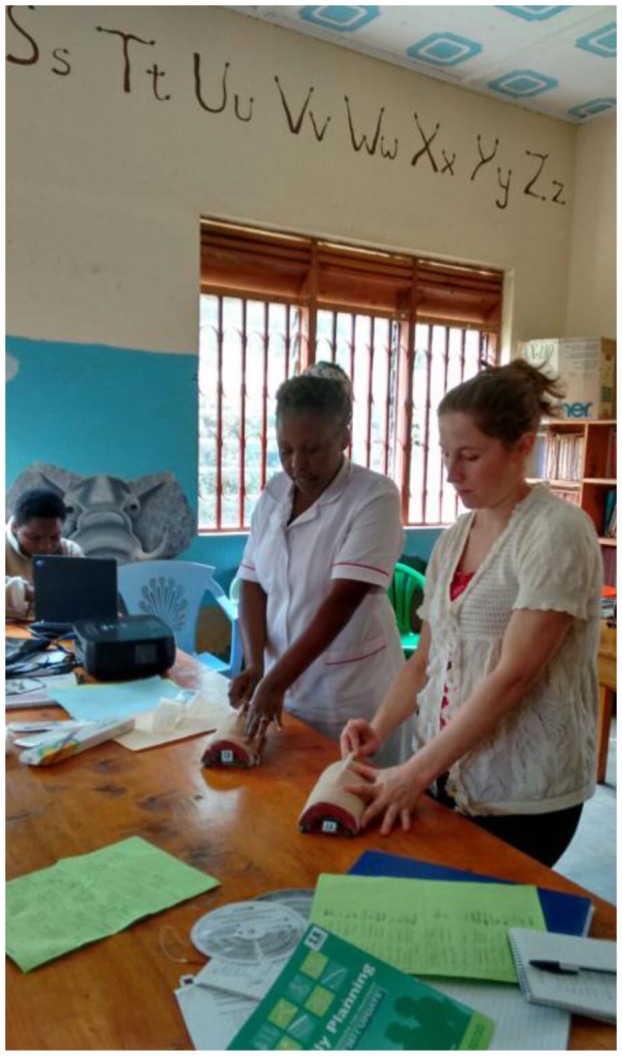
The author and Sister Damari demonstrating implant insertion to Level 2 trainees.

In April 2015, Sister Damari Kagaza, an enrolled nurse went to Bwindi Community hospital for basic Level 1 and higher Level 2 training. Sister Damari was enthused and inspired by this training and helped to run the training at Kisiizi hospital. 68 members of staff at Kisiizi trained to a basic level, by attending 4 h of training. This included medical, midwifery, surgical and nursing staff as well as support staff. Even more staff attended the first session which was an overview of why family planning matters to us all, including managers, religious leaders, the dentist and even the tailor (see Figure 1).

**Figure 2.**
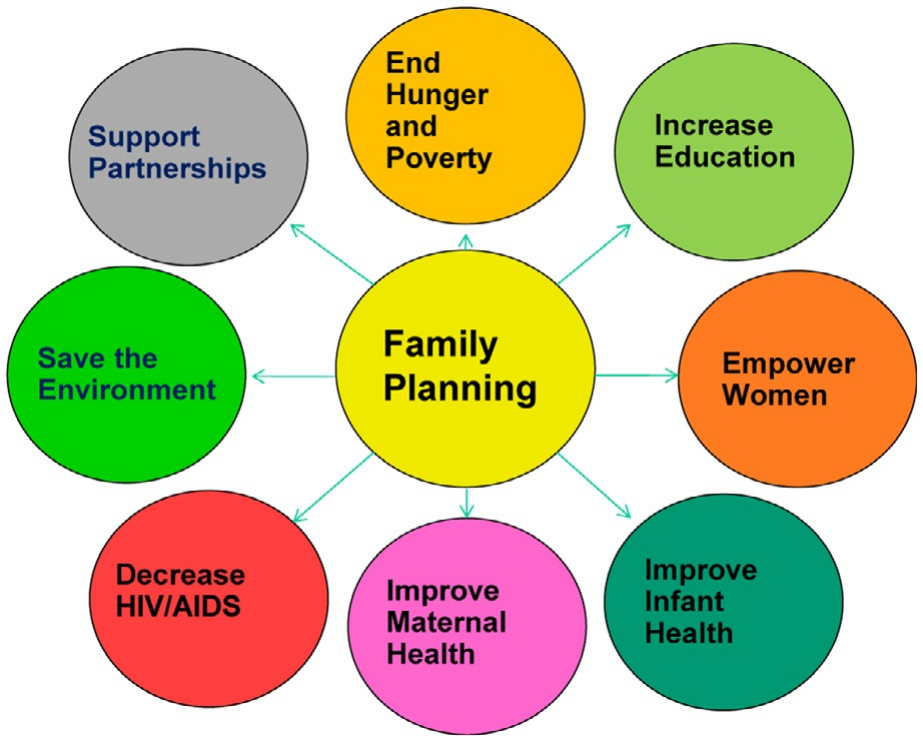
Family planning and the Millennium development goals.[10]

**Figure 3.**
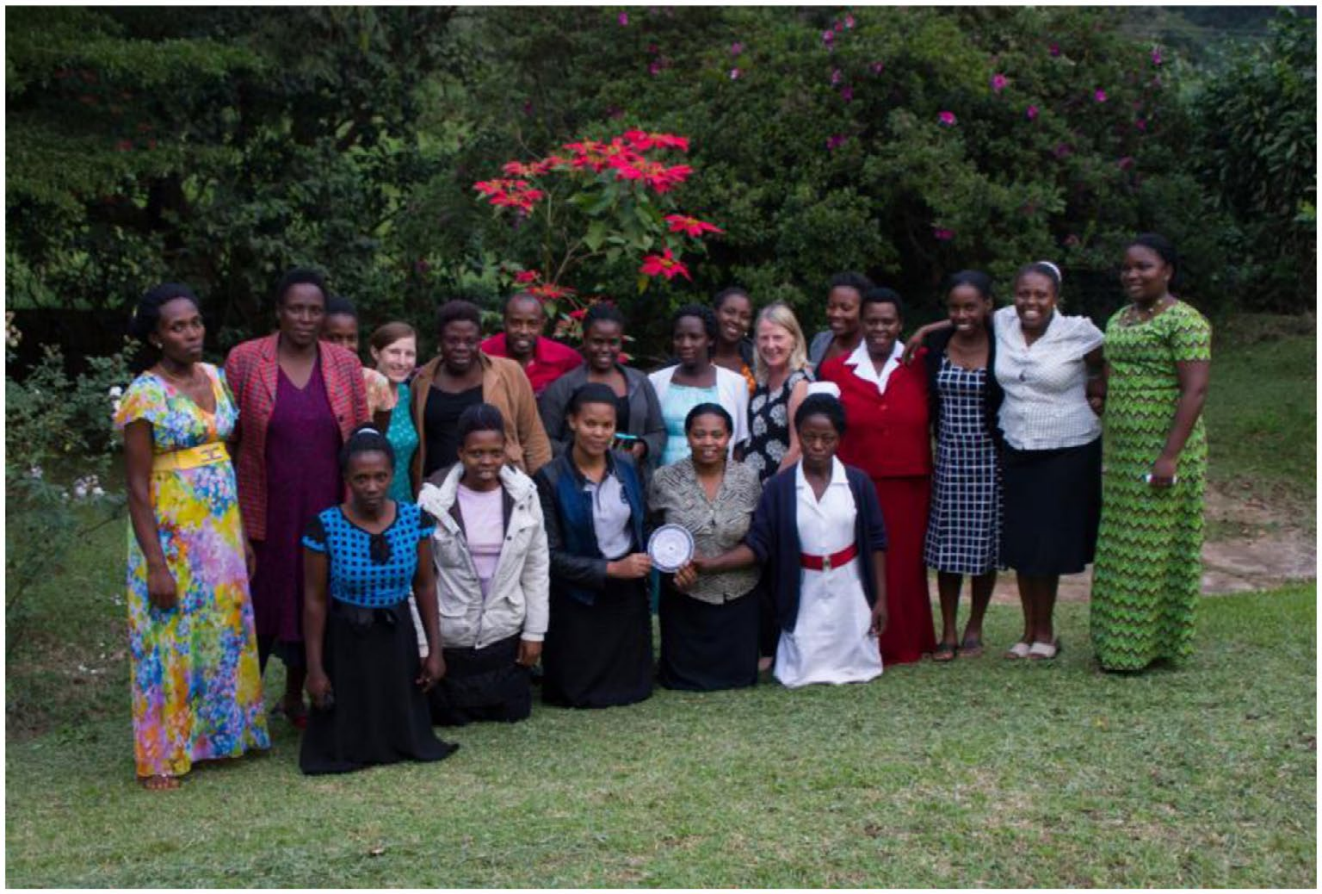
Candidates successfully passing Level 2 training.

A further 32 staff have been trained to Level 2 higher level. These were nurses from maternity unit, antenatal clinic, surgical ward, medical ward and diploma student nurses. In their own unpaid time, they attended 30 h of training over 5 days, including sessions on anatomy and physiology, counselling, sensitive scenarios, talking to teenagers and men, cervical cancer and in-depth knowledge of family planning methods. They learned the practical skills necessary for intrauterine coil insertion and implant insertion, and had a chance to practice on real patients. They all had to give a community education talk, and all managed to pass a rigorous written exam. Feedback was gained from Levels 1 and 2 candidates as to their post-course confidence, and their feedback on the content and delivery of the course, which will shape future training (see Figure 2 and 3).

**Table:** 

Some feedback from the training
Level 1 feedback from a 19-year-old female student nurse “the course of family planning has been interesting and encouraging to everyone, whether married or unmarried, so as to encourage everyone who has heard to teach all the people in the nation in order to prevent maternal & infant mortality”
Level 2 feedback from a 28-year-old male surgical nurse “the course was so wonderful and interesting. Above all it was educating. Thank you very much you people and may the Lord God bless you. This is just the beginning”

## Discussion

The barriers to development of family planning at Kisiizi include religious beliefs, fear of side effects, concern that family planning can cause HIV and cancer, fear of subsequent subfertility and the high cost of services (for example, it costs 20000 shillings for an implant, around £5). Following the USHAPE activity the management at Kisiizi have agreed to support family planning by making many methods free to members of the health insurance scheme and significantly reducing the costs of coils and implants.

The candidates in Level 2 training demonstrated that they now have the skills and confidence to teach in the community. Level 2 candidates have taken the initiative further, with support from the USHAPE team to organise their own men’s engagement sessions. They have put together a patient education film in the local language, Rukiga, for use in outpatients and the HIV clinics.[8]

There are areas which need development, such as engaging with the youth. In a predominantly Christian environment, abstinence is emphasised, and there are concerns that more comprehensive sex education and contraception will increase sexual activity. However, there is strong evidence to the contrary. Research shows youth who receive comprehensive sex education are NOT more likely to become sexually active, increase sexual activity or experience negative sexual health outcomes.[8] More work is needed with creating the demand for services out in the community, as well as provision of family planning in community outreach settings.

Our cascade model of training, involves training Ugandan USHAPE trainers and ownership of the project by local staff. The USHAPE initiative clearly demonstrates that timely input of short-term projects with a longer term endeavour is helpful. We are ambitious that by following a public health model,[9] focusing on service delivery to all who need it that it has the potential to be scaled up to other hospitals, health centres and communities.

## Governance

State which governance group oversaw this work (e.g. ethics committee, NHS organisation, university). Some group must oversee quality assurance if data are generating from real-life situations.

## Disclosure statement

No potential conflict of interest was reported by the authors.
